# Efficient and Reliable Identification of Probabilistic Cloning Attacks in Large-Scale RFID Systems

**DOI:** 10.3390/mi16080894

**Published:** 2025-07-31

**Authors:** Chu Chu, Rui Wang, Nanbing Deng, Gang Li

**Affiliations:** 1 The College of Computer Science, Sichuan Normal University, Chengdu 610101, China; 20241393056@stu.sicnu.edu.cn (R.W.); 2021110711@stu.sicnu.edu.cn (N.D.); 2 The School of Information and Software Engineering, University of Electronic Science and Technology of China, Chengdu 610054, China; ligangpm@uestc.edu.cn

**Keywords:** radio frequency identification, probabilistic cloning attack, identification reliability, time efficiency, block mechanism, missing tags

## Abstract

Radio Frequency Identification (RFID) technology is widely applied in various scenarios, including logistics tracking, supply chain management, and target monitoring. In these contexts, the malicious cloning of legitimate tag information can lead to sensitive data leakage and disrupt the normal acquisition of tag information by readers, thereby threatening personal privacy and corporate security and incurring significant economic losses. Although some efforts have been made to detect cloning attacks, the presence of missing tags in RFID systems can obscure cloned ones, resulting in a significant reduction in identification efficiency and accuracy. To address these problems, we propose the block-based cloned tag identification (BCTI) protocol for identifying cloning attacks in the presence of missing tags. First, we introduce a block indicator to sort all tags systematically and design a block mechanism that enables tags to respond repeatedly within a block with minimal time overhead. Then, we design a superposition strategy to further reduce the number of verification times, thereby decreasing the execution overhead. Through an in-depth analysis of potential tag response patterns, we develop a precise method to identify cloning attacks and mitigate interference from missing tags in probabilistic cloning attack scenarios. Moreover, we perform parameter optimization of the BCTI protocol and validate its performance across diverse operational scenarios. Extensive simulation results demonstrate that the BCTI protocol meets the required identification reliability threshold and achieves an average improvement of 24.01% in identification efficiency compared to state-of-the-art solutions.

## 1. Introduction

With the rapid expansion of the Internet of Things (IoT) technology, Radio Frequency Identification (RFID) technology has been adopted in a wide range of applications, including logistics, healthcare, and inventory control [1,2,3]. A typical RFID system consists of one or more readers and multiple RFID tags. Each tag contains a unique 96-bit identifier (ID) and transmits stored data when triggered by radio waves from the reader [4,5,6]. The reader can communicate with tags based on specific requirements and transfer the collected data to a back-end server for further processing and analysis. Compared with traditional two-dimensional codes, RFID offers several advantages, such as faster identification speeds, a longer identification distance, and support for non-line-of-sight communication [7,8,9].

Despite the advantages of RFID systems, their widespread adoption has raised significant security concerns, among which cloning attacks pose a major threat. Specifically, attackers employ specialized devices to extract data from legitimate tags and produce cloned versions. These clone tags can occupy the same time slot as their corresponding genuine tags, generating interference that causes signal collisions [10,11]. As a result, the RFID reader fails to receive or distinguish valid information, thereby disrupting the system’s normal operation. For example, in the retail industry, clone tags can be used to manipulate inventory management systems, causing inventory discrepancies and the circulation of counterfeit products. Furthermore, attackers can generate clone tags using data harvested from employee ID cards, which enables unauthorized individuals to bypass access control systems, alter corporate transaction records, or exfiltrate sensitive information.

Existing works, however, remain inadequate in addressing the intricate challenges of the problem under consideration. Specifically, most methods assume an ideal scenario where clone tags launch attacks with a 100% probability [12,13,14]. This assumption allows probabilistic cloning attacks to easily evade detection, leading to a high false-negative rate. On the other hand, although the identifying probabilistic cloning attacks (IPCA) protocol attempts to address probabilistic attacks by incorporating probabilistic models, it fails to account for the presence of missing genuine tags, as cloned tags can exploit these absences to mimic normal system behavior, thereby compromising efficiency and accuracy. For instance, to evade detection risks, attackers can adopt probabilistic cloning attacks, where clone tags transmit signals in an intermittent and random manner to mimic genuine tag behavior [15,16,17]. Moreover, the loss of genuine tags due to movement or theft is prevalent in many scenarios, further complicating the detection of cloning attacks [18]. Therefore, the efficient and reliable identification of probabilistic cloning attacks is of paramount importance, motivating us to develop a more robust and accurate protocol.

In this paper, we propose the block-based cloned tag identification (BCTI) protocol for the efficient and reliable identification of probabilistic cloning attacks in the presence of missing tags. The identification process of BCTI consists of three phases. First, the reader broadcasts the required parameters to all tags and precomputes the block index for each genuine tag. Next, based on these computations, the reader constructs and broadcasts a block vector to sort each genuine tag into its corresponding block for subsequent verification. Finally, leveraging the presence and absence characteristics of genuine tags, the reader adjusts the actual block order and requires tags in each block to respond multiple times sequentially to identify cloning attacks. The main contributions of this paper are as follows:

- We consider a more comprehensive and realistic scenario, where some genuine tags are subjected to probabilistic cloning attacks and there exist missing genuine tags, and develop an efficient and reliable solution to address the compounded detection challenges.
- We propose a block-based tag sorting mechanism that organizes tags into distinct blocks for targeted sequential interrogation, thereby reducing the overhead of repeated parameter and vector broadcasts by the reader.
- We develop a multi-response verification method that uses differentiated interrogation lengths for blocks containing present or missing tags to reduce execution time, and introduces a superposition mechanism for concurrent verification of cloned tags across both block types to improve time efficiency.
- We theoretically analyze and optimize critical parameters to enhance both the identification accuracy and time efficiency of our protocol, validating its performance through extensive numerical experiments.

The structure of the remaining sections is organized as follows. Section 2 reviews related research works. Section 3 presents the system model and defines the problem to be addressed. Section 4 details the proposed BCTI protocol and provides a comprehensive analysis of parameter optimization. Section 5 evaluates the performance of the BCTI protocol through extensive experiments and simulations. Section 6 discusses the impact of detection errors and capture effects on the BCTI protocol in practical scenarios. Finally, Section 7 concludes this paper and outlines future research directions.

## 2. Related Works

Clone tags mimicking legitimate ones can bypass traditional RFID monitoring systems, leading to information leakage and economic losses [19,20]. Meanwhile, missing tags can further complicate monitoring, escalating the need for robust detection mechanisms against cloning attacks. With the rapid advancement of RFID technology, research efforts have focused on two key domains: missing tag identification and cloning attack identification. In this section, we present a detailed review of recent progress in these domains.

### 2.1. Missing Tag Identification

Missing tag identification aims to efficiently detect tags that exit RFID systems. In [21], the authors proposed the slot filter-based missing tag identification (SFMTI) protocol, which uses indicator vectors to convert collision slots into singleton slots and skips the detection of non-singleton slots. As research advanced, heterogeneous tag scenarios were incorporated into the analysis. The authors of [18] addressed cloned tag challenges and introduced three protocols: concurrent missing tag identification (CMTI), group-based missing tag identification (GMTI), and collision-reconciled missing tag identification (RMTI). These protocols partition tags into cloned and uncloned subsets, querying each subset to identify missing tags. However, their assumption of a 100% cloned tag attack probability may limit real-world applicability. To achieve multi-group detection efficiency, the authors of [22] proposed the accurate and expeditious multiple-group missing tag detection (AEMD) protocol, which leverages Manchester encoding and indicator vectors to rapidly detect missing tags across categories. A limitation is its focus on missing tag events rather than exhaustive tag enumeration. To handle unknown tag interference, the authors of [23] developed the priority-based strategy for missing unknown tag identification (PMUTI) protocol, which prioritizes silencing known tags via hierarchical mechanisms, sequentially marks unknown tags, and uses filter vectors for missing tag identification. Based on the demand for the priority identification of key tags, the authors of [24] proposed the verification and selection (VAST) protocol and its enhanced version (E-VAST), which employ an iterative verification-selection framework with ON-OFF keying modulation and pre-selection to directly identify target key tags.

In short, existing works on missing tag identification (MTI) in RFID systems have evolved from single-scenario detection to addressing complex environments involving cloned tags, multi-category tags, and unknown tags. These methodologies offer valuable insights for verifying the presence of genuine tags within various application scenarios.

### 2.2. Cloning Attack Identification

Cloning attack identification aims to determine which tags in RFID systems are subject to cloning attacks. To identify the specific IDs of all cloned tags, the authors of [14] proposed a slotted broadcast-friendly cloned-tag identification (S-BID) protocol. S-BID is based on the multi-round identification process of the frame-slotted Aloha protocol, in which tags randomly select slots to respond with random numbers, and the reader predicts slot states using genuine tag IDs. If a predicted singleton slot results in a collision, the genuine tag assigned to that slot is considered cloned, and its ID is treated as the ID of the cloned tag. To address scenarios involving known and unknown genuine tag IDs, the authors of [25] developed the Blocking Detection Polling (BDP) and Fast Blocking Detection (FBD) protocols, which are based on tree-based structures. The authors of [13] addressed the challenge of newly added tags and proposed a hybrid protocol integrating Aloha Filtering (AF) and Poll-and-Listen (P&L) mechanisms, in which AF efficiently filters out interference from new tags, while P&L accurately identifies cloning attacks by sequentially polling the IDs of genuine tags. Considering that most works assume a 100% clone tag attack probability, the authors of [26] proposed the identifying probabilistic cloning attacks based on segmentation (S-IPCA) technique, which requires the genuine tag in each singleton time slot to respond multiple times until either the time slot becomes a collision time slot or the set threshold for the number of responses is reached. To perform repeated verification, S-IPCA must spend considerable time broadcasting multiple vectors.

In summary, most existing protocols rely on the idealized assumption of a 100% clone tag attack probability, which significantly limits their applicability in real-world scenarios. While the IPCA protocol attempts to identify probabilistic cloning attacks, its time efficiency could be improved. Furthermore, in the presence of missing tags, a common issue in RFID applications, existing methods lack effective mechanisms for identifying cloning attacks, leading to a notable decline in detection accuracy. To address these limitations, we propose the BCTI protocol, which employs blocking and superposition mechanisms to continuously verify genuine tags in different states, thereby enabling the efficient identification of cloning attacks.

## 3. System Model and Problem Formulation

### 3.1. System Model

**Figure 1:**
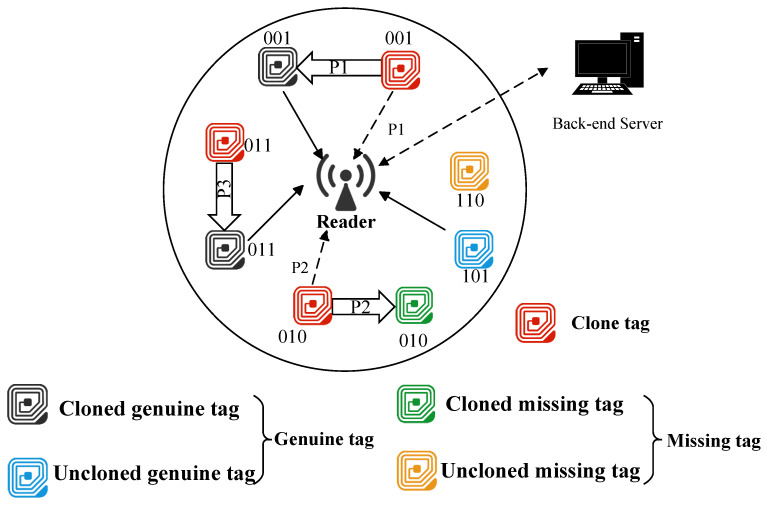
Example of a probabilistic cloning attack.

As shown in Figure 1, the considered large-scale RFID system comprises a back-end server, a reader, and multiple tags. Within the reader’s coverage area, tags transmit their data in response to the reader’s command, and the reader then forwards the data to the back-end server for further processing. Each tag has a unique 96-bit ID and is preconfigured with a hash function H(·). This configuration aligns with a fundamental premise of hash-based protocols (HEPs) [13,21,26], which necessitates that both tags and readers operate under a shared hash function for proper functionality. In our considered problem, tags are categorized into five distinct types:

- Uncloned genuine tag: The original tag that is legally registered within the system and has not been cloned or removed from the system.
- Cloned genuine tag: The original tag that has been cloned due to the illegal acquisition of key information.
- Uncloned missing tag: The uncloned genuine tag that has been removed from the system’s identification range due to theft or management errors.
- Cloned missing tag: The genuine tag that has been cloned and removed from the system’s identification range.
- Clone tag: An unauthorized entity that copies genuine tag data, posing a security threat via cloning attacks.

### 3.2. Communication Model

To increase slot utilization and reduce tag collisions, we adopt the frame-slotted Aloha (FSA) protocol [27] for communications between the reader and tags. In FSA, time is divided into multiple frames, and each frame consists of a predetermined number of slots. At the beginning of a frame *i*, the reader sends a query command containing the frame length $F_{i}$ and a random seed $R_{i}$. Based on this command and specified rules, each tag obtains a slot index by calculating $j=H\left(ID,R_{i}\right)\;\mathrm{mod}\;F_{i}$ and responds with specified data. The slots in the frame are categorized by their states into three types: (1) empty slots with no tag response; (2) singleton slots with only a single tag response; and (3) collision slots with multiple tag responses. Slots can also be classified by length as follows: (1) tag slots, denoted by $t_{\mathrm{tag}}$, for transmitting a 96-bit ID or data; (2) long-response slots, denoted by $t_{l}$, for multi-bit data transmission; and (3) short-response slots, denoted by $t_{s}$, for one-bit data transmission. Following parameter configurations from prior studies [28,29,30], we set $t_{\mathrm{tag}}=2.4$ ms, $t_{l}=0.8$ ms, and $t_{s}=0.4$ ms.

### 3.3. Attack Model

In open RFID systems, cloning attacks refer to unauthorized entities that replicate data from legitimate RFID tags to create counterfeit ones. The cloning attack model addressed in this paper for RFID systems has the following characteristics:

1. Probabilistic Attacks: Each cloned genuine tag may be subject to probabilistic attacks from its corresponding clone version, with the attack probability $P_{y}$.
2. Attack Impact: When a genuine tag is queried, its clone version may respond in the same time slot with probability $P_{y}$, causing signal collisions that prevent the reader from receiving valid information.
3. Missing Impact: For a missing tag, the reader receives no response when no cloning attack occurs, but if an attack occurs on a cloned missing tag, the reader will receive data from the clone version with probability $P_{y}$.
4. Attack Uncertainty: The reader lacks prior knowledge of which tags are cloned and the individual attack probabilities of each clone tag. This information gap introduces substantial unpredictability into attack dynamics, complicating the efficient identification of cloned tags.

The schematic diagram in Figure 1 illustrates the probabilistic cloning attack model in an open RFID system, where each tag is assumed to have a 3-bit ID and is categorized into five types as described in Section 3.1. In this system, tags with IDs 001, 011, and 010 have been cloned. When the reader queries surrounding tags and requests their responses, clone tags select the same time slot as their corresponding genuine tags and launch attacks with probabilities $P_{1}$, $P_{2}$, and $P_{3}$, respectively. Based on the tag types, the reader may encounter different response scenarios. As shown in Figure 1, the clone tag with ID 011 does not attack, allowing the reader to receive information from the genuine tag. Since the genuine tag with ID 010 is missing, the reader instead receives responses from its clone version. Conversely, the clone tag with ID 001 launches an attack, causing a collision by responding simultaneously with the genuine tag and thus preventing the reader from receiving valid information.

### 3.4. Problem Formulation

In open RFID systems, it is of utmost importance to efficiently and accurately identify cloned tags under reliability constraints. Let *M* denote the set of all genuine tags, which includes the subset *C* of cloned tags and the subset *Q* of missing tags. The missing tags can be identified by the initial execution of protocols such as SFMTI [21] for tag identification. In our considered problem, two key parameters are defined: an attack probability threshold $P_{t}\in \left(0,1\right)$ and a minimum required identification reliability level α∈(0,1). Additionally, each clone tag launches attacks on its corresponding genuine tag with a random probability $P_{y}\in \left[P_{t},1\right)$. In general, the objective of this study can be summarized as follows: given prior knowledge of the total number of genuine tags |M| and a required identification reliability level α, design a protocol to maximize identification accuracy and ensure that the number of successfully identified cloned tags is at least α·|C|. The notations used throughout this paper are systematically summarized in Table 1.

## 4. BCTI Protocol Design

### 4.1. Description of BCTI

This section elaborates on the block-based cloned tag identification (BCTI) protocol, which identifies cloned tags in open RFID systems through block sorting and continuous interrogation. BCTI consists of three phases. During the vector construction phase, the reader generates multiple indicator vectors to assign corresponding block numbers to each present and missing genuine tag. During the tag sorting phase, tags select actual blocks to respond according to the order of block numbers in the vectors and specific commands. When all unverified tags are sorted in multiple rounds of the first two phases, the tag verification phase begins. During this phase, the reader analyzes the continuous responses across various blocks to determine the cloning status of each tag and adjusts its response block as needed. The detailed process of BCTI is described below.

#### 4.1.1. Vector Construction Phase

During this phase, the reader broadcasts parameters and constructs indicators to sort each tag. Specifically, in the *i*-th round of the phase, the reader generates an indicator vector *V*, where each element in *V* corresponds to a block number. For each unverified genuine tag $t_{j}$, the reader predicts its selected block index $b_{j}$ using $H\left({ID}_{j},R_{i}\right)\;\mathrm{mod}\;N_{i}$, where ${ID}_{j}$ is stored in the back-end server and $N_{i}$ is the number of blocks in round *i*. When a block is mapped by only one present tag or one missing tag, the reader sets $\begin{array}{c} V_{b_{j}} \end{array}=11$ or $\begin{array}{c} V_{b_{j}} \end{array}=10$, respectively, and $V_{b_{j}}=0$ otherwise.

**Figure 2:**
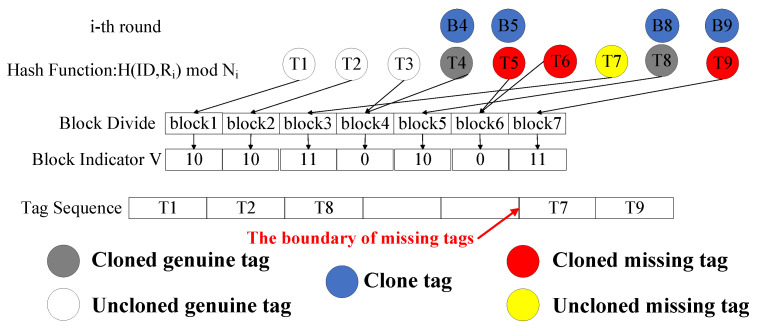
Example of the vector construction phase and the tag sorting phase in the *i*-th round.

An example of this phase is presented in Figure 2, where blocks 1, 2, 3, 5, and 7 are selected by only one present or one missing genuine tag, so the reader sets V[1]=V[2]=V[5]=10 and V[3]=V[7]=11. This vector, along with a random seed $R_{i}$, the number of present genuine tags $g_{p}$, and the block number $N_{i}$, is broadcast to the tags by the reader.

#### 4.1.2. Tag Sorting Phase

During this phase, tags determine their block order and actual response blocks based on the received information. Specifically, after each tag receives the parameters broadcast by the reader, it calculates $b_{j}=H\left({\mathrm{ID}}_{j},R_{i}\right)\;\mathrm{mod}\;N_{i}$ to determine the corresponding indicator index and records its value. When $V[b_{j}]=0$, the tag participates in the next round of the vector construction phase. When $V[b_{j}]=1$, the tag calculates $s=b_{j}-\phi_{1}+\zeta_{1}$ as its block order, where $\phi_{1}$ and $\zeta_{1}$ are the number of 11 s plus 0 s before $V[b_{j}]$ in the current round and the number of 10 s in the previous rounds, respectively. When $V[b_{j}]=11$, the tag calculates $s=b_{j}-\phi_{2}+g_{p}+\zeta_{2}$ as its block order, where $\phi_{2}$, $\zeta_{2}$, and $g_{p}$ are the number of 10 s plus 0 s before $V[b_{j}]$ in the current round, the number of 11 s in the previous rounds, and the number of all present tags, respectively. The reader executes the first two phases round by round until all tags have obtained a block number.

**Figure 3:**
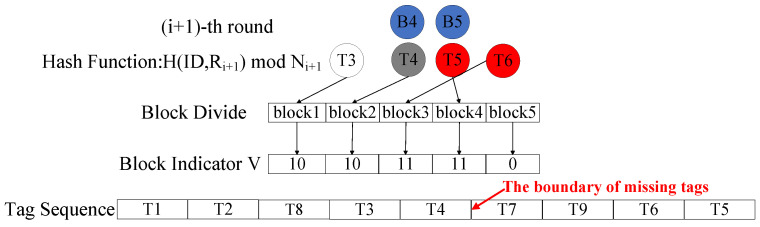
Example of the vector construction phase and the tag sorting phase in the i+1-th round.

As shown in Figure 2, we assume that the system contains five present tags and four missing tags. In the first round, since $T_{8}$ finds that its corresponding value in *V* is 10, $\phi_{1}=2$, and $\zeta_{1}=0$, it calculates s=5−2+0=3. Similarly, $T_{1}$ and $T_{2}$ calculate s=1−0+0=1 and s=2−0+0=2. On the other hand, since $T_{9}$ finds that its corresponding value in *V* is 11, $\phi_{2}=5$, $\zeta_{2}=0$, and $g_{p}=5$, it calculates s=7−5+5+0=7. Similarly, $T_{7}$ calculates s=6. In the second round as illustrated in Figure 3, the reader broadcasts *V*, $\zeta_{1}=3$, and $\zeta_{2}=2$ to unverified tags. Then, $T_{3}$ calculates s=1−0+3=4, $T_{4}$ calculates s=2−0+3=5, $T_{5}$ calculates s=4−2+2+5=9, and $T_{6}$ calculates s=3−2+2+5=8. After that, all tags are assigned a block number.

#### 4.1.3. Tag Verification Phase

During this phase, the reader verifies whether tags have been affected by cloning attacks based on their response status. Specifically, the reader first assesses the cloned tag ratio ρ. When ρ meets the pre-set threshold condition, the superposition verification strategy is executed; otherwise, the one-by-one verification method is adopted. When implementing the superposition strategy, let $s_{p}$ denote the block index of a present tag and $s_{m}$ the index of a missing tag block that can be superimposed with it, where $s_{m}$ and $s_{p}$ satisfy $s_{m}=\left|M\right|-\left|Q\right|+s_{p}$ and $s_{m}\leq \left|M\right|$ (here, |M| represents the total number of blocks, and |Q| is the number of missing tag blocks). The reader then sends an instruction to make the tag with index $s_{m}$ respond synchronously in the actual block corresponding to $s_{m}-\left|M\right|+\left|Q\right|$ along with the present tag at index $s_{p}$, transmitting the multi-bit information $t_{l}$. Concurrently, the reader initializes an |M|-bit status vector *E* with all elements set to 0. During *K* consecutive interrogation of block $s_{p}$, if no collision is detected, the reader determines that neither of the superimposed tags has been cloned, and sets $E_{s_{p}}=1$; if a collision occurs during any interrogation, since the cloning status cannot be determined, $E_{s_{p}}$ remains 0 and the relevant tags return to their original indices $s_{m}$ and $s_{p}$ for subsequent verification. After completing the verification of all superimposed blocks, the reader broadcasts the status vector *E* to instruct tags to update their states accordingly. Specifically, tags with $E_{s}=1$ enter an inactive state and no longer respond to subsequent interrogation, while tags with $E_{s}=0$ adjust their indices to $s=s-\theta_{s}$ (where $\theta_{s}$ denotes the number of 1 s preceding index s in *E*). If the number of collision-free verifications $K^{\prime }$ within the superimposed blocks is less than *K*, which indicates insufficient verification, the reader conducts one-by-one consecutive interrogations for each tag block. In this method, tags with original indices satisfying s<|M|−|Q| are required to reply with the long response $t_{l}$, while the remaining tags should reply with the short response $t_{s}$. During the queries, the detection of a collision in the $t_{l}$ response or the presence of a signal in the $t_{s}$ response indicates a cloned tag, whereas no collision or signal after $K-K^{\prime }$ consecutive queries confirms that the tag has not been cloned.

**Figure 4:**
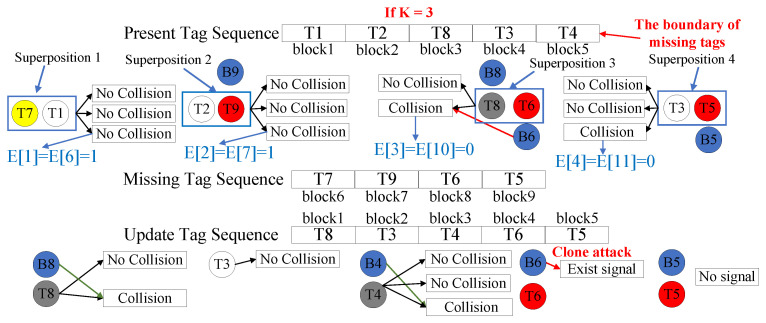
Superposition verification in the verification phase.

**Figure 5:**
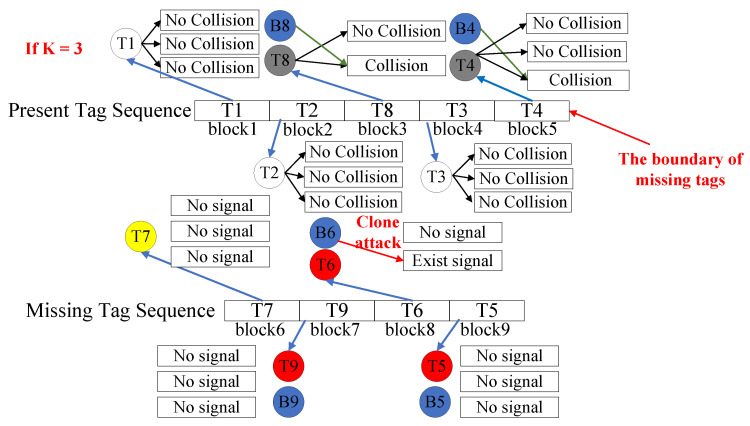
One-by-one verification in the verification phase.

An example of the verification phase is shown in Figure 4. Suppose the maximum number of interrogations is K=3, and the current ratio of cloned tags ρ satisfies the threshold condition. Since |M|=9 and |Q|=4, tag $T_{9}$ will be superimposed for verification with tag $T_{1}$ in block 6−9+4=1. Similarly, $T_{9}$, $T_{6}$, and $T_{5}$ are superimposed into blocks 2 to 4, respectively. The reader then queries the superimposed blocks in sequence and generates the vector E. As no collision signals are detected in the three interrogations to block 1, the reader identifies that $T_{1}$ and $T_{7}$ are non-cloned and sets E(1)=E(6)=1. Analogously, $T_{2}$ and $T_{9}$ are also identified as non-cloned, so E[2]=E[7]=1. Furthermore, since collisions are detected in blocks 3 and 4, the reader cannot determine the cloning status of the tags and then records the number of collision-free verifications completed in each block as $K^{\prime }=1$ and $K^{\prime }=2$, and sets E[3]=E[10]=E[4]=E[11]=0. Upon receiving *E*, the non-inactivated tags update their block indices. For example, the block index of $T_{3}$ is updated to 4−2=2. The reader then continues to execute one-by-one verifications in each block for $K-K^{\prime }$ times. As no collision occurs in the remaining one verification of $T_{3}$, it is identified as non-cloned, while $T_{8}$ and $T_{4}$ are found to be cloned in their remaining two and three verifications, respectively. On the other hand, the missing tags $T_{6}$ and $T_{5}$ are identified as cloned and non-cloned, respectively, because signals are detected for $T_{6}$ and no signals are present for $T_{5}$ in the remaining verifications. Figure 5 also illustrates the process of directly executing the one-by-one verification method. For blocks corresponding to present tags, the reader detects whether collisions occur in the long responses $t_{l}$ of tags within each block; for blocks corresponding to missing tags, it checks for the presence of signals in the short responses $t_{s}$ of tags. With a maximum of 3 verifications, the reader identifies that $T_{8}$, $T_{4}$, and $T_{6}$ are cloned, while all other tags remain uncloned. Compared with one-by-one verification, superposition verification can reduce the number of detections by 7.

### 4.2. Parameter Optimization

In this section, we theoretically analyze the setting of three key parameters in the BCTI protocol: the number of consecutive queries *K* within a block, the number of blocks per round $N_{i}$, and the threshold of cloned tag ratio ρ for superposition verification. This analysis aims to satisfy the reliability requirements for identification while maximizing identification efficiency.

#### 4.2.1. Optimal K-Value Analysis

We first analyze the setting of the *K* value to achieve the required identification reliability with the minimum number of interrogations in each block. During at most *K* verification attempts, BCTI can identify cloned tags in two scenarios: detecting a collision when querying a present genuine tag, or receiving a response when querying a missing genuine tag. Recall that $P_{y}$ is the probability of a clone tag launching an attack and α is the identification reliability requirement. The probability $P_{j}$ that the cloned tag can be correctly identified can be calculated as

(1) $$1-{\left(1-P_{y}\right)}^{K}=P_{j}.$$

Since each clone tag launches attacks with a random probability $P_{y}\in \left[P_{t},1\right)$, the expected number of successfully identified cloned tags can satisfy the following inequality:

(2) $$\begin{array}{cc} \sum_{j\in C}P_{j} & =\sum_{j\in C}1-{\left(1-P_{y}\right)}^{K}\\ \; & \geq |C|\cdot [1-{\left(1-P_{t}\right)}^{K}]. \end{array}$$

By combining (1) and (2), we can obtain the following inequalities concerning *K* to ensure that at least α·|C| cloned tags can be successfully identified:

(3) $$\begin{array}{cc} \left|C\right|\cdot \left[1-{\left(1-P_{t}\right)}^{K}\right] & \geq \alpha \cdot |C|\\ K & \geq \frac{\ln\left(1-\alpha \right)}{\ln\left(1-P_{t}\right)} \end{array}$$

Since *K* is an integer, the maximum number of verifications for tags in each block can be set as follows:

(4) $$K^{*}=\left\lceil \frac{\ln\left(1-\alpha \right)}{\ln\left(1-P_{t}\right)}\right\rceil .$$

#### 4.2.2. Optimal N-Value Analysis

Recall that $N_{i}$ is the total number of blocks, and let $m_{i}$ be the number of unassigned genuine tags in the *i*-th round. The probability that a block is exclusively selected by a single unverified genuine tag can be calculated as

(5) Pi=mi1×1Ni×1−1Nimi−1≈miNi×e−miNi.

Then, the expected number of blocks $B_{i}$ that can be assigned to tags in the *i*-th round is

(6) $$B_{i}=N_{i}\times P_{i}=m_{i}e^{-\frac{m_{i}}{N_{i}}}.$$

Let $T_{read}$ denote the execution time of the reader in the *i*-th round. $T_{read}$ consists of the time required to broadcast the block indicator *V* and other parameters to all tags. Based on the construction method of *V*, we can calculate $T_{\mathrm{read}}^{i}$ as

(7) $$T_{\mathrm{read}}^{i}=t_{\mathrm{tag}}\times \left\{1+\left[\frac{\left(P_{i}\times 2N_{i}\right)+\left[\left(1-P_{i}\right)\times N_{i}\right]}{96}\right]\right\},$$

where $t_{tag}$ is the time to transmit 96-bit data. Combining (6) and (7), the average time ϵ to assign a block successfully can be calculated as

(8) $$\begin{array}{cc} \epsilon & =\frac{T_{\mathrm{read}}^{i}}{B_{i}}\\ \; & =\frac{t_{\mathrm{tag}}\left(1+\frac{m_{i}}{96}e^{-\frac{m_{i}}{N_{i}}}+\frac{N_{i}}{96}\right)}{m_{i}e^{-\frac{m_{i}}{N_{i}}}}. \end{array}$$

By differentiating (8) with respect to $N_{i}$, the following expression can be derived:

(9) $$\frac{d\epsilon }{dN_{i}}=\frac{t_{\mathrm{tag}}\left(-96m_{i}+N_{i}^{2}-m_{i}N_{i}\right)e^{\frac{m_{i}}{N_{i}}}}{96m_{i}N_{i}^{2}}.$$

To determine the optimal value of $N_{i}$, we set (9) equal to 0. According to (9), the simplified expression for the optimal number of blocks $N_{i}$ is given by

(10) $${N_{i}}^{2}-N_{i}m_{i}-96m_{i}=0.$$

By solving (10), the optimal value of $N_{i}$ is

(11) $$N_{i}=\frac{m_{i}\pm \sqrt{m_{i}\left(m_{i}+384\right)}}{2}.$$

Although two optimal solutions exist for $N_{i}$, the positive value is typically selected in practical applications and is expressed as

(12) $$N_{i}=\frac{m_{i}+\sqrt{m_{i}\left(m_{i}+384\right)}}{2}.$$

#### 4.2.3. Superposition Strategy Analysis

Let ρ denote the ratio of cloned tags in the system. According to the verification process of BCTI, a superposition verification can be completed within the same time slot under the following scenarios: (1) neither the superposed missing tag nor the present tag has been cloned; and (2) among the two superposed tags, there is one or two cloned tags, yet none of these clone tags launches an attack. Then, we define *t* as the time saving and initialize t=0. When superposition verification is performed, if no cloning attack occurs, the execution time $t_{s}$ is saved, whereas if a cloning attack occurs, $t_{l}$ is wasted. Thus, the expression for *t* is derived as

(13) t=−O1ts+O2(1+Pt)2tl−O2[1−(1+Pt)2]ts=−O31−1+Pt22ts+O3[1−1−1+Pt22]tl.

where $O_{1}$, $O_{2}$, and $O_{3}$ are the proportions where the superimposed two tags are both uncloned, only one is cloned, and both are cloned, respectively. Recall that $t_{s}=0.4$ ms and $t_{l}=0.8$ ms in this paper, we have

(14) $$\begin{array}{cc} t & =-{\left(1-\rho \right)}^{2}t_{s}+2\rho \left(1-\rho \right)\left[\frac{1+P_{t}}{2}t_{l}-\left(1-\frac{1+P_{t}}{2}\right)t_{s}\right]+\rho^{2}t_{l}\left[1-\frac{3}{2}{\left(1-\frac{1+P_{t}}{2}\right)}^{2}\right]\\ \; & =-0.3{\left(1+P_{t}\right)}^{2}\rho^{2}+1.2\left(1+P_{t}\right)\rho -0.4. \end{array}$$

To save time, we require t<0, which means that $0.3{\left(1+P_{t}\right)}^{2}\rho^{2}-1.2\left(1+P_{t}\right)\rho +0.4>0$. Therefore, we can calculate $\Delta =0.96{\left(1+P_{t}\right)}^{2}$. Then, the roots can be obtained as $c_{1}=\frac{2(3-\sqrt{6})}{3(1+P_{t})}$ and $c_{2}=\frac{2(3+\sqrt{6})}{3(1+P_{t})}$. Since $P_{t}\in \left(0,1\right)$, we have $c_{2}>1$. Given ρ∈[0,1], the valid solution is $\rho <\frac{2(3-\sqrt{6})}{3(1+P_{t})}$. Therefore, when the ratio of cloned tags is lower than $\frac{2(3-\sqrt{6})}{3(1+P_{t})}$, we superpose the missing tag and the present tag in the same block to accelerate the identification rate.

## 5. Performance Evaluation

In this section, we evaluate the performance of our BCTI protocol against the state-of-the-art IPCA and S-IPCA protocols [26]. Here we modify S-IPCA and IPCA based on the tag verification rules in BCTI to enable them to identify the cloning status of missing genuine tags. We employ two verification strategies: BCTI_sup, which applies the superposition strategy to each block initially and switches to one-by-one verification upon detecting a collision, and BCTI_one, which verifies the tags in each block sequentially and continuously without superposition.

### 5.1. Performance Settings

The simulation setting follows the EPCglobal C1G2 standard [27], defining the tag-to-reader transmission rate as 40–460 kb/s and the reader-to-tag rate as 26.7–128 kb/s. Since the comparison involves IPCA and S-IPCA, we adopt the parameters from [26], setting the time for the tag’s short response as $t_{s}=0.4$ s, the tag’s long response as $t_{l}=0.8$ s, and the reader’s transmission of 96 bits to all tags as $t_{tag}=2.4$ s. Through the aforementioned configuration, this paper establishes a simulation framework for cloned tag identification in RFID systems, enabling a systematic evaluation of the BCTI protocol’s performance characteristics. Simulation data are generated based on these predefined parameters, with the subsequent analysis focusing on two core metrics: computational efficiency (i.e., execution time) and actual identification reliability. We conduct comparative analyses across four key factors: the number of genuine tags, the number of cloned tags, the number of missing tags, and the cloning attack probability threshold. To ensure results reliability, each simulation is run 100 times by default, with the average outcomes reported from independent trials.

### 5.2. Impact of Genuine Tags

To investigate the impact of the number of genuine tags on the BCTI protocol, we vary the number of genuine tags |M| from 1000 to 10,000 in steps of 1000, set the number of cloned tags to 0.1×|M| and the number of missing tags to 0.3×|M|, and fix the attack probability threshold $P_{t}$ at 0.5.

**Figure 6:**
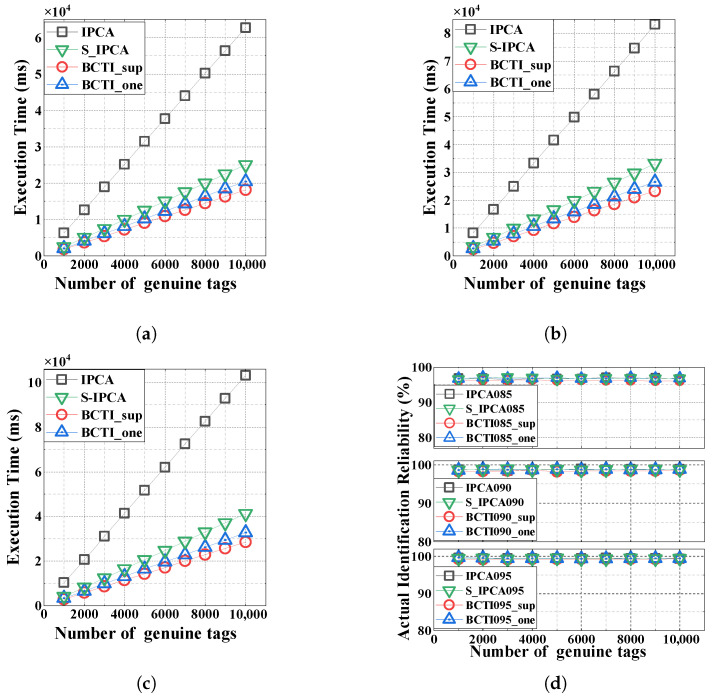
Impact of the number of genuine tags on the total execution time and actual identification reliability. (**a**) α=0.85; (**b**) α=0.90; (**c**) α=0.95; (**d**) identification reliability.

Figure 6 depicts the execution time and identification reliability across three reliability levels (α=0.85,0.90, and 0.95). We can observe in Figure 6a–c that the execution time increases linearly with the number of genuine tags, where BCTI outperforms the other two methods under all reliability requirements. This superiority stems from BCTI’s block mechanism, which reduces the reader broadcast time, and its strategy of setting distinct response lengths for present and missing tags during continuous interrogation to minimize response overhead. For example, when the number of genuine tags is 10,000 and α=0.95, BCTI_sup and BCTI_one achieve execution times of 28.49 s and 32.74 s, representing reductions of 30.85% and 20.53% compared to S-IPCA’s 41.20 s. Notably, BCTI_sup further enhances verification efficiency via superposition verification in scenarios with low cloned tag ratios, thus saving more time than BCTI_one under the same conditions.

As shown in Figure 6d, all four methods exhibit similar actual identification reliabilities. The proposed BCTI protocol maintains a stable actual identification reliability across varying numbers of genuine tags |M|, consistently achieving levels significantly higher than the target reliability α.

### 5.3. Impact of Cloned Tags

To investigate the impact of the number of cloned tags on the BCTI protocol, we set the number of genuine tags |M| to 5000 and fix the number of missing tags |Q| to 1500 while varying the number of cloned tags |C| from 500 to 2500 in 500-step increments. The attack probability threshold $P_{t}$ is consistently set at 0.5 across all experimental conditions.

**Figure 7:**
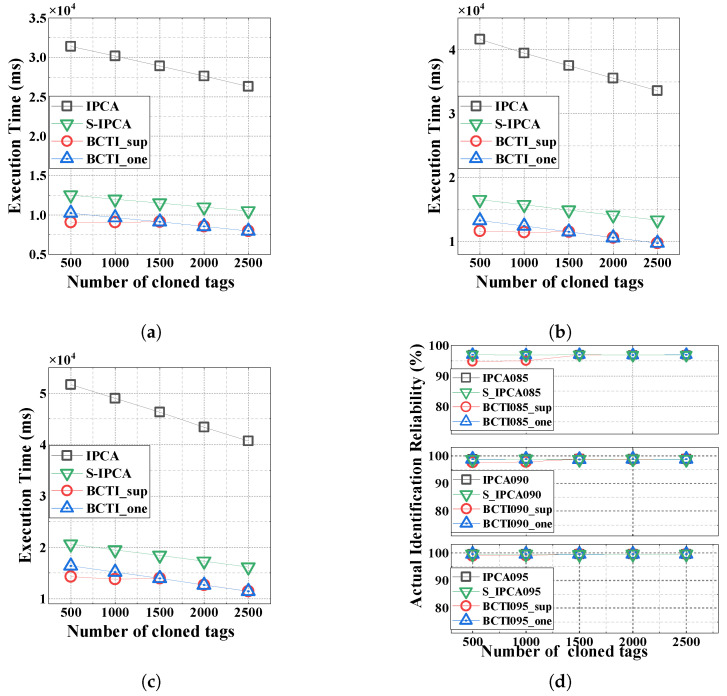
Impact of the number of cloned tags on the total execution time and actual identification reliability. (**a**) α=0.85; (**b**) α=0.90; (**c**) α=0.95; (**d**) identification reliability.

Figure 7 depicts the execution time and identification reliability across three reliability levels (α=0.85,0.90, and 0.95). We can observe that as the number of cloned tags increases, the average verification count per genuine tag decreases, thereby reducing the execution time for all methods. The simulation results in Figure 7a–c reveal that the proposed BCTI protocol outperforms comparative methods in execution time. For example, at α=0.90 with 500 cloned tags, S-IPCA exhibits an execution time of 16.56 s, while BCTI_sup and BCTI_one achieve 11.68 s and $\begin{array}{c} 13.32 \end{array}$ s, respectively, representing reductions of 29.47% and $\begin{array}{c} 19.57 \end{array}\%$ compared to S-IPCA under the same conditions.

For the actual identification reliability, as shown in Figure 7d, the proposed BCTI protocol ensures identification accuracy well above the target reliability α under different numbers of cloned tags |C|. Note that when |C|=2000, BCTI_sup switches from superposition verification to one-by-one verification because the ratio of cloned tags does not meet the threshold, resulting in slight changes in the actual identification reliability and execution time.

### 5.4. Impact of Missing Tags

To investigate the impact of the number of missing tags on the BCTI protocol, we fix the number of genuine tags |M| at 5000, the number of cloned tags |C| at 1000 and vary the number of missing tags |Q| from 500 to 2500 in 500-step increments while keeping all other settings consistent with those described in Section 5.2.

**Figure 8:**
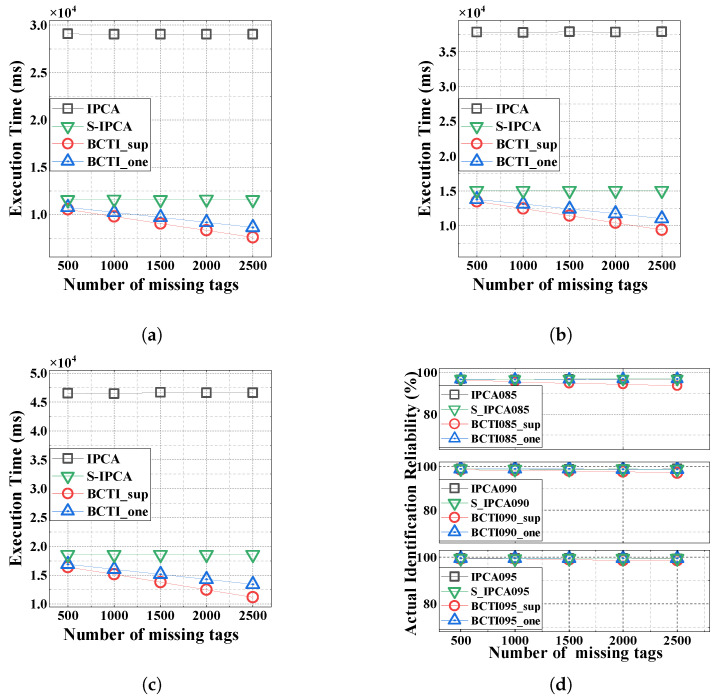
Impact of the number of missing tags on the total execution time and actual identification reliability. (**a**) α=0.85; (**b**) α=0.90; (**c**) α=0.95; (**d**) identification reliability.

Figure 8 shows the execution time and identification reliability across three reliability levels (α=0.85,0.90, and 0.95). We can observe in Figure 8a–c that as the number of missing tags increases, the execution time of BCTI decreases accordingly, and it consistently outperforms the other methods. The underlying reason for this is that a higher number of missing tags leads to more blocks where only short responses are required to verify tag cloning. Moreover, BCTI_sup enables superposition verification of more missing and genuine tags, thereby further shortening the execution time by leveraging combined responses. For example, when the number of missing tags is 2500 and α=0.85, the execution times of BCTI_sup and BCTI_one are 7.55 s and 8.62 s, while that of S-IPCA is 11.57 s under the same conditions. Compared with S-IPCA, BCTI_sup and BCTI_one reduce the execution time by 34.75% and 25.50%, respectively.

As shown in Figure 8d, the identification reliabilities of BCTI_sup and BCTI_one are still much higher than the required α. We can also observe that as the number of missing tags increases, BCTI_sup executes more superposition verification, thereby causing a slight decrease in identification reliability. On the other hand, BCTI_one maintains the same performance as the other methods.

### 5.5. Impact of the Cloning Attack Probability Threshold

To investigate the impact of the cloning attack probability threshold on the BCTI protocol, we fix the number of genuine tags |M| at 5000, the number of cloned tags |C| at 1000, and vary the attack probability threshold $P_{t}$ from 0.1 to 0.9 in 0.1-step increments while keeping all other experimental parameters consistent with those described in Section 5.2.

**Figure 9:**
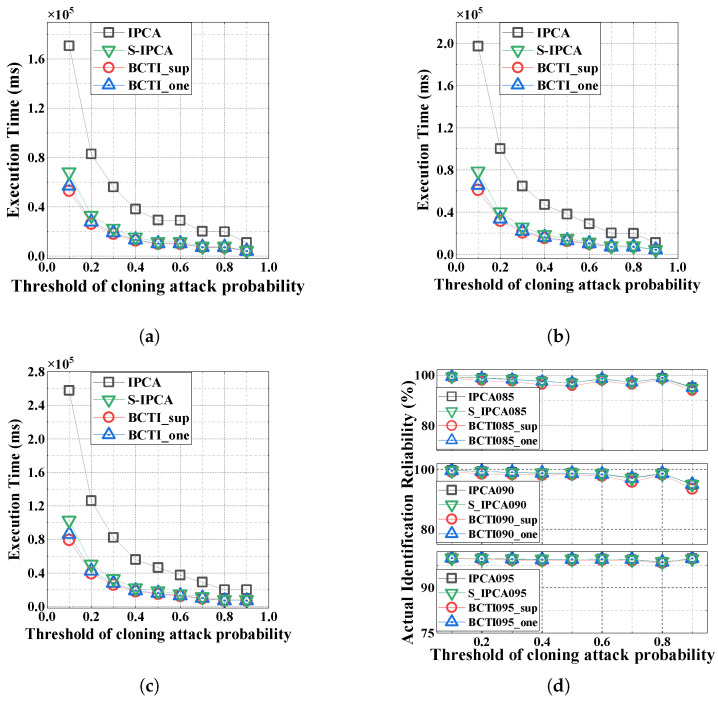
Impact of the cloning attack probability threshold on the total execution time and actual identification reliability. (**a**) α=0.85; (**b**) α=0.90; (**c**) α=0.95; (**d**) identification reliability.

Figure 9 shows the execution time and identification reliability across three reliability levels (α=0.85,0.90, and 0.95). Similarly to Section 5.2, the average verification count per genuine tag decreases as the cloning attack probability threshold increases, thus reducing the total execution time. Our BCTI protocol remains faster than the other two methods, as illustrated in Figure 9a–c. For example, when the cloning attack probability threshold is 0.4 and α=0.90, S-IPCA requires 18.67 s, whereas BCTI_sup and BCTI_one achieve execution times of 15.21 s and 16.09 s, thus reducing the execution time by 18.53% and 13.82%, respectively, compared to S-IPCA.

As shown in Figure 9d, as the cloning attack probability threshold increases, the required verification time *K*, which should be an integer, decreases accordingly, leading to some fluctuations in the actual identification reliability.

## 6. Discussion

In this section, we analyze the impact of a practical environment on the proposed BCTI protocol. Specifically, two critical factors dominate: detection errors and capture effects [31]. In passive RFID systems, environmental interference can distort tag backscatter signals, leading to detection errors that can transform a singleton slot into an empty one or a collision slot into a singleton/empty slot. On the other hand, backscattered signal intensity attenuates with increasing tag–reader distance. When tags at different distances respond to the reader simultaneously, the stronger signal may override weaker ones, inducing the capture effect that transforms an original collision slot into a singleton slot. Note that we exclude transmission bit errors from consideration, since in indoor environments with reader-tag distances <10 m and the signal-to-noise ratio of the passive RFID >15 dB, the bit error probability remains below $10^{-6}$ [32].

**Figure 10:**
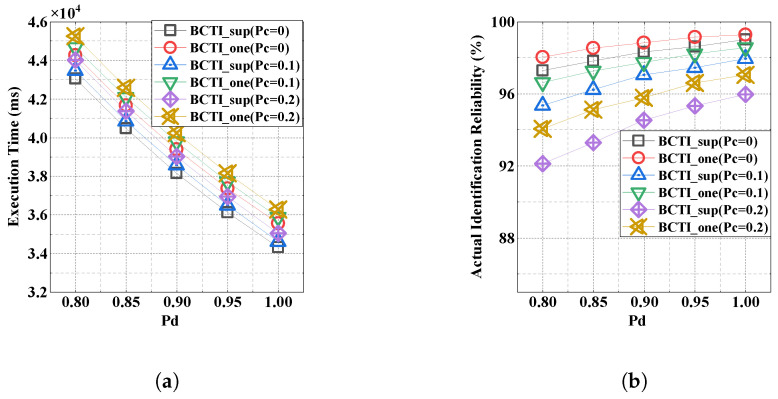
The impact of detection errors and capture effects on BCTI. (**a**) Execution time. (**b**) Actual identification reliability.

Figure 10a investigates the impact of environmental factors on identification performance, with the parameters set to α=0.9, |M|=5000, $P_{t}=0.2$, |Q|=0.1×|M|, and $\left|C\right|=\begin{array}{c} 0.2 \end{array}\times \left|M\right|$. We denote the detection errors and capture effect as $P_{d}$ and $P_{c}$, respectively, and specify their values as $P_{d}=\left[0.80,0.85,0.90,0.95,1.00\right]$ and $P_{c}=\left[0,0.1,0.2\right]$. We can observe in Figure 10a that the detection errors generate numerous empty slots during tag verification, while the capture effects reduce the collision detection probability, thereby allowing some cloning attacks to evade detection and prolonging the execution time. Compared with BCTI_one, BCTI_sup achieves a shorter execution time, but because of a higher tag density per slot, its superposition strategy makes identification reliability more susceptible to detection errors and capture effects. To address this, BCTI_sup can allocate moderate additional time by increasing the verification rounds for each block.

## 7. Conclusions

In this paper, we proposed the block-based cloned tag identification (BCTI) protocol to address the challenges of identifying probabilistic cloning attacks in RFID systems with missing tags. Unlike existing methods, BCTI introduces a block indicator to sequence tags systematically and a block mechanism that enables repeated tag responses within slots with minimal time overhead. In addition, the superposition strategy is designed to optimize tag responses and further reduce the execution time. Through in-depth analysis of tag response patterns, BCTI can accurately identify cloning attacks and mitigate interference from missing tags. We theoretically analyzed and optimized critical parameters to balance identification accuracy and efficiency and validated the protocol through extensive simulations. The results show that BCTI outperforms state-of-the-art methods, achieving an average 24.01% improvement in identification efficiency while exceeding reliability thresholds. In future research, we will further investigate multi-reader scenarios, in which the probability of cloning attacks may vary randomly within a certain period, and consider conducting experiments in multiple-tag scenarios.

## Figures and Tables

**Table 1:** Notations used in this paper.

Notation	Description
M/C/Q	The set of genuine tags/cloned genuine tags/missing tags
$N_{i}$	The number of blocks in the *i*-th round
$m_{i}$	The number of unassigned genuine tags in the *i*-th round
α	Required identification reliability
$P_{y}$	The attack probability of each clone tag
$P_{t}$	The attack probability threshold
*V*	The block indicator
$K^{*}$	The maximum number of verifications for tags in each block
$T_{\mathrm{read}}^{i}$	The broadcast time of the reader in the *i*-th round

## Data Availability

The data presented in this study are available on request from the corresponding author.
